# Single-incision versus standard multi-incision laparoscopic colectomy in patients with malignant or benign colonic disease: a systematic review, meta-analysis and assessment of the evidence

**DOI:** 10.1186/s12893-016-0187-5

**Published:** 2016-10-18

**Authors:** Anne Catharina Brockhaus, Stefan Sauerland, Stefan Saad

**Affiliations:** 1 Department of Medical Biometry, Institute for Quality and Efficiency in Health Care (IQWiG), Cologne, Germany; 2 Institute for Health Economics and Clinical Epidemiology, University of Cologne, Cologne, Germany; 3 Department of Non-Drug Interventions, Institute for Quality and Efficiency in Health Care, Cologne, Germany; 4 Department of General, Abdominal, Vascular and Thoracic Surgery, Academic Hospital University Cologne, Cologne, Germany

**Keywords:** Single port, Mini-invasive, Laparoscopy, Surgery, Colon

## Abstract

**Background:**

Single-incision laparoscopic colectomy (SILC) requires only one umbilical port site and (depending on technique) a specimen extraction site.

The aim of this study was the assessment of the available evidence for the comparison of SILC to conventional multi-port laparoscopic colectomy (MLC) in adult patients, in whom elective colectomy is indicated because of malignant or benign disease. First, previous meta-analyses on this topic were assessed. Secondly, a systematic review and meta-analysis of randomised controlled trials, was performed.

**Methods:**

Electronic literature searches (CENTRAL, MEDLINE and EMBASE; up to March 2016) were performed. Additionally, we searched clinical trials registries and abstracts from surgical society meetings. For meta-analysis, risk ratios (RR) or mean differences (MD) with 95 % confidence intervals were calculated and pooled. The quality of previous meta-analyses was evaluated against established criteria (AMSTAR) and their reported results were investigated for consistency.

**Results:**

We identified 6 previous meta-analyses of mostly low methodological quality (AMSTAR total score: 2 − 5 out of 11 items). To fill the evidence gaps, all these meta-analyses had included non-randomised studies, but usually without assessing their risk of bias. In our systematic review and meta-analysis of randomised controlled trials exclusively, we included two randomised controlled trials with a total of 82 colorectal cancer patients. There was insufficient evidence to clarify whether SILC leads to less local complications (RR = 0.52, 95 % CI 0.14 − 1.94) or lower mortality (1 death per treatment group). Length of hospital stay was significantly shorter in the SILC group (MD = -1.20 days, 95 % CI -1.95 to -0.44). One of the two studies found postoperative pain intensity to be lower at the first day. We also identified 7 ongoing trials with a total sample size of over 1000 patients.

**Conclusion:**

The currently available study results are too sparse to detect (or rule out) relevant differences between SILC and MLC. The quality of the current evidence is low, and the additional analysis of non-randomised data attempts, but does not solve this problem. SILC should still be considered as an experimental procedure, since the evidence of well-designed randomised controlled trials is too sparse to allow any recommendation.

## Background

During conventional multi-port laparoscopic colonic resection (MLC), the camera and surgical instruments are inserted through 4 − 5 trocars. The resected colon part is extracted by an additional minilaparotomy (i.e. low Pfannenstiel or midline incision). Laparoscopic colonic surgery increasingly became the new standard for colorectal resection [1, 2]. There is evidence that incisional hernias are less frequent using the total-laparoscopic approach instead of open abdominal surgery [3]. For caesarean section, wound length was found as a risk factor for surgical-site complications [4].

Therefore, newer approaches and advances of the minimal invasive surgery aim to minimise the total length of incisions even further, which in turn may reduce the morbidity of the abdominal wall, such as wound pain, wound infection and hernia formation. This implies the expectation of a faster recovery in the early postoperative phase. One way to achieve this aim is to minimise the number of incisions used. Single-incision laparoscopic colectomy (SILC) uses only one umbilical port site [5, 6]. However, the likely limitations of SILC include an additional learning curve and advanced laparoscopic skill requirements [7], because triangulation is missing, when all instruments are oriented intraabdominally in the same direction [8, 9].

There are several meta-analyses published [10–15] comparing SILC to MLC, none of which included randomised controlled trials exclusively, but predominantly observational studies such as case-matched studies. Most of these reviews noted substantial heterogeneity in some of their outcomes [10, 11, 13], which might reflect the differences in study design, surgical technique, patient selection, postoperative care or even the incomplete learning curve among the different studies. The potential bias of the results due to the low quality of the included studies was also addressed by several reviews [10–12]. It is important to assess the efficacy and safety of SILC by preparing this systematic review based on only RCTs. Including only RCTs minimises the heterogeneity and potential bias mentioned above that might be introduced into the analysis by the inclusion of observational studies.

The aim of this work is the assessment of the available evidence. This includes an investigation of the methodological quality and results of previously published meta-analyses comparing SILC to MLC in adult patients. Furthermore and as previously specified in the protocol to this systematic review [16], we compared SILC to MLC in adult patients, in whom elective colectomy is indicated because of malignant or benign disease, by performing a systematic review and meta-analysis of randomised controlled trials.

## Methods

Previously published meta-analyses on this topic (i.e. SILC vs. MLC) were systematically identified from the same literature searches as described below. Meta-analyses were eligible, if they examined SILC in the treatment of malignant or benign diseases of colon or rectum. The methodological quality of these meta-analyses was assessed by using AMSTAR (‘A Measurement Tool to Assess Systematic Reviews’), which contains 11 single items and give a maximum score of 11 points [17]. The appraisal of meta-analyses was independently done by two reviewers.

 We conducted this systematic review according to a pre-specified protocol [16]. The protocol describes the surgical procedures studied, the eligible patient groups, as well as the pre-specified methods (i.e. criteria for considering studies for this review, search strategy, data collection and analysis). Thus, unless stated otherwise, the present systematic review was performed according to the protocol [16].

The search was conducted from 2008 to March 2016. Electronic literature searches were performed in the databases CENTRAL, MEDLINE and EMBASE. For the search in two clinical trial registries, the following terms were used: ‘single-incision laparoscopic colectomy’, ‘single-port laparoscopic colectomy’, ‘single AND colectomy’, ‘single AND incision AND colon’, ‘single AND incision AND colectomy’, ‘single AND port AND colon’, ‘single AND port AND colectomy’, ‘transumbilical AND colectomy’, ‘transumbilical AND colon’, ‘notes AND colectomy’, ‘notes AND colon’. In addition, a manual search of several potentially relevant systematic reviews and meta-analyses on this topic [10–15, 18] was carried out to identify additional trials.

Eligible studies were selected independently by two authors according to the previously specified criteria (i.e. RCT, SILC and MLC as intervention, adult patients, in whom elective colectomy was indicated because of either malignant or benign disease). Primary outcomes were previously defined as local complications (intra- and postoperative events) and mortality. Secondary outcomes were defined as conversion rate to laparoscopic, hand-assisted laparoscopic or open surgery, estimated blood loss, operative time, number of patients with R0 resection, tumour-free resections or both, number of lymph nodes harvested, postoperative pain intensity, general complications, resumption of bowel function, length of hospital stay, quality of life or fatigue, cosmetic results and disease-free survival.

We contacted authors of potentially eligible studies to obtain any missing information. The study by Poon et al. [19] presented their results as median, but they kindly provided mean and standard deviations for their reported continuous outcomes upon request. Therefore, no imputation of missing data was relevant. The risk of bias assessment was performed using the criteria described in the Cochrane Handbook for Systematic Reviews of Interventions [20].

We intended to explore reasons for heterogeneity (Chi2 test with significance being set at *P* value < 0.05) in the studies using subgroup and sensitivity analysis, but this was not possible due to the low number of studies. This is also the reason, why the assessment of potential publication bias using a funnel plot would not have been meaningful. In cases of substantial statistical heterogeneity we did not pool the results. Analysis was conducted using Review Manager Version 5.3 [21].

## Results

### Assessment of previous meta-analyses

A total of 8 systematic reviews comparing SILC to MLC were found [10–15, 18, 22]. Two reviews [18, 22] also included case series and case reports and were therefore excluded from detailed assessment here.

Thus, we investigated the methodological quality of 6 systematic reviews, all of which also included meta-analyses [10–15]. According to the AMSTAR instrument [17, a systematic review is well done, when all the Items on the checklist have been fulfilled. As summarized in Table 1, all of the systematic reviews complied with only two to five of the 11 items. Major issues were a missing a priori published protocol, presentation of the search strategy or additional searches such as trial registries or conference proceedings and a missing quality assessment of the included studies. All systematic reviews checked whether heterogeneity existed for the included studies, but nearly all the systematic reviews pooled the studies regardless of the presence or absence of statistical heterogeneity. Furthermore, there were several discrepancies among the systematic reviews with regard to the inclusion of primary studies (Table 2). Therefore, the results and conclusions of these reviews may be affected by substantial methodological bias stemming from either the primary studies or the systematic review itself.

**Table 1:** Assessment of methodological quality of systematic reviews by using the AMSTAR instrument

Systematic review	I1	I2	I3	I4	I5	I6	I7	I8	I9	I10	I11	AMSTAR Total score
Podda 2016 [15]	n	?	y	y	n	y	n	n	n	y	y	5
Markar 2014 [14]	n	?	?	y	n	y	n	n	n	y	y	4
Maggiori 2012 [10]	n	?	n	y	n	y	y	y	n	n	y	5
Zhou 2012 [11]	n	?	?	n	y	y	y	n	n	y	y	5
Li 2012 [12]	n	?	n	n	n	y	n	n	y	y	n	3
Yang 2012 [13]	n	?	?	n	n	y	n	n	n	y	n	2

y: yes, the criteria are met; n: no, the criteria are not met; ?: can’t answer

I1: Was an ‘a priori’ design provided?

I2: Was there duplicate study selection and data extraction?

I3: Was a comprehensive literature search performed?

I4: Was the status of publication (i.e. grey literature) used as an inclusion criterion?

I5: Was a list of studies (included and excluded) provided?

I6: Were the characteristics of the included studies provided?

I7: Was the scientific quality of the included studies assessed and documented?

I8: Was the scientific quality of the included studies used appropriately in formulating conclusions?

I9: Were the methods used to combine the findings of studies appropriate?

I10: Was the likelihood of publication bias assessed?

I11: Was the conflict of interest included?

**Table 2:** Characteristics of assessed systematic reviews

Author (Year)	Types of resections included	Included studies	Conclusion on SILC
Podda et al. [15]	Right-sided, left-sided or total colectomy (including ileocecal resection)	30 studies: 2 RCTs [19, 23], 28 observational studies [24–51]	“safe and feasible”
Markar et al. [14]	Right-sided, left-sided or total colectomy	34 studies: 2 RCTs [19, 23], 32 observational studies [24–48, 52–58]	“similar short-term clinical and oncological outcomes”
Maggiori et al. [10]	Right-sided, left-sided or total colectomy	15 studies: 0 RCTs, 15 observational studies [24–26, 30–32, 36–39, 41, 43, 46, 47, 52]	“feasible and safe”
Zhou et al. [11]	Right-sided, left-sided or total colectomy (including ileocecal resection)	14 studies: 1 RCT [23], 13 observational studies [24, 26, 30, 32, 34, 37, 38, 41, 43, 46, 52, 55, 56]	“safe, feasible, and oncologically efficient”
Li et al. [12]	unclear	11 studies: 1 RCT [23], 10 observational studies [24–26, 30, 31, 37, 41, 46, 52, 55]	“short-term results similar”
Yang et al. [13]	Right-sided or left-sided colectomy	15 studies: 1 RCT [23], 14 observational studies [24–26, 30–32, 34, 37, 41, 43, 44, 46, 47, 59]	“similar safety and efficacy”

### Systematic review and meta-analysis of randomised controlled trials

We retrieved 686 records (531 different publications after duplicates removed) through database searching and 10 additional records through other sources (society meetings and study register), of which 529 records clearly did not meet the inclusion criteria and were therefore excluded. Four records were only available as conference abstracts [60–64]. We contacted the authors, who either did not respond or confirmed that their study is still ongoing [63, 64]. One study was only available as study protocol [65] and the other five records were only listed as study registration without any full-text available.

**Fig. 1:**
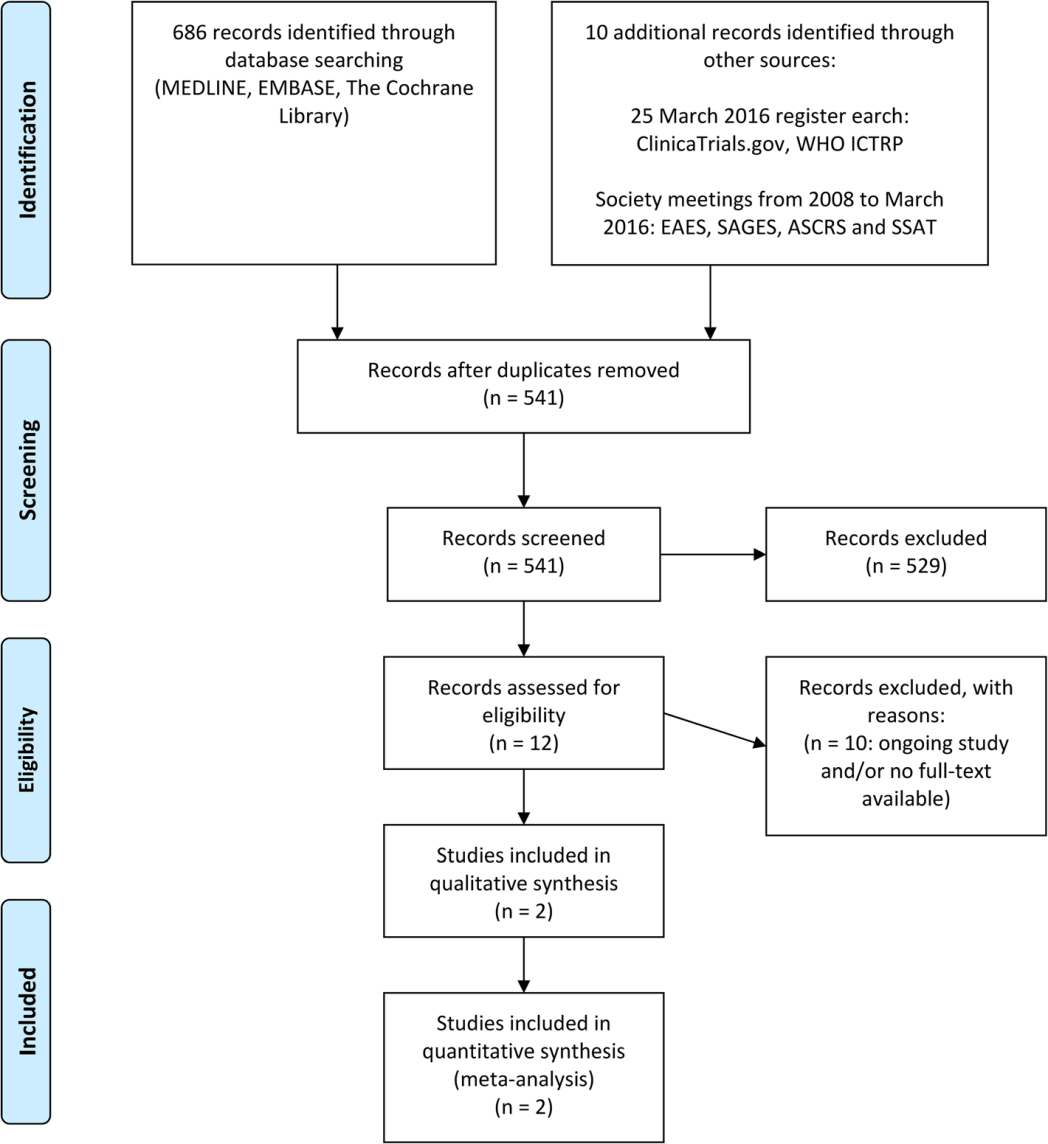
Literature search and study flow

Two full texts randomised trials [19, 23] were retrieved, evaluated in detail and included in the present review. We did not identify any additional study from screening the reference lists of included studies, potentially-relevant systematic reviews on the same topic, online trial registries or congress proceedings. The result of this search was illustrated in the flow diagram (Fig. 1).

### Study characteristics

We identified two randomised controlled trials [19, 23] were included in this systematic review. None of the included studies investigated benign diseases. Thus the following results are derived only for malignant diseases. In the study by Poon et al. [19], specimen extraction was done through the umbilicus, whereas Huscher et al. [23] also used a colpotomy in female patients. While the patients in the study by Poon et al. [19] received an enhanced recovery program, this was not mentioned in the studies by Huscher et al. [23]. The characteristics of the included studies are summarised in Table 3.

**Table 3:** Baseline characteristics of studies included in the meta-analysis

Author, year (country)	No. of patients	Age in years	Gender (M/F)	BMI in kg/m^2^	ASA (1, 2, 3)	Type of colectomy (left/right)	Cancer stage (1, 2, 3)	
Huscher et al. [26], 2012 (Italy)	32	70 ± 11	15 / 17	not reported	8 (25 %),	SILC: 8/8	SILC:	MLC:
	(16 vs. 16)	(mean ± SEM)			15 (47 %),	MLC: 10/6	5 (31 %),	4 (25 %),
					9 (28 %)		7 (44 %),	9 (56 %),
							4 (25 %)	3 (19 %)
Poon et al. [16], 2012 (China)	50	SILC: 67 (37-83)^a^	SILC: 14 / 11	SILC: 23.2 (16.9-28.8)^a^	6 (12 %),	SILC: 17^b^/8	SILC:	MLC:
	(25 vs. 25)	MLC: 67 (57-81)^a^	MLC: 18 / 7	MLC: 23.6 (16.5-28.2)^a^	37 (74 %),	MLC: 16^b^/9	8 (32 %),	5 (20 %),
					7 (14 %)		7 (28 %),	4 (16 %),
							6 (24 %)	12 (48 %)

*M* Male, *F* Female, *BMI* body mass index, *ASA* American Society of Anaesthesiologists, *SILC* Single-incision laparoscopic colectomy, *MLC* multi-incision laparoscopic colectomy

^a^Data are given as median with range ^b^including anterior resection and sigmoidectomy

We assessed the overall risk of bias of the study by Huscher et al. [23] as high, due to an unclear random sequence generation (exact method of sequence generation not specified), missing blinding of the participants, personnel and outcome assessors, as well as a missing study registration, definition of a primary outcome variable or pre-specified sample size (high risk due to possibly selective reporting).

We assessed the overall risk of bias of the study by Poon et al. [19] as low, because all of the assessed risks of bias were low, except for the documented surgical experience, which was not reported in the publication. The authors kindly informed us that all the conventional laparoscopic colectomies were operated by a team of four surgeons (experience of more than 50 MLC cases), of whom two surgeons (experience of more than 10 SILC) performed all the single incision laparoscopic. We assessed the risk of bias due to a possibly not completed learning curve as unclear.

**Fig. 2:**
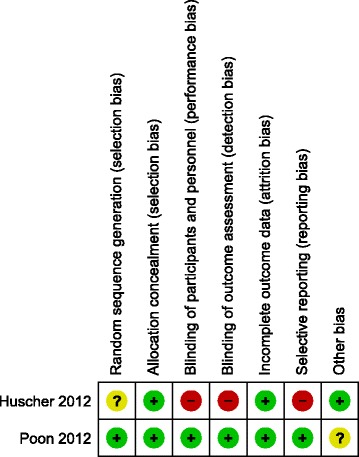
Risk of bias summary: review authors’ judgements about each risk of bias item for each included study

The results of the risk of bias assessment are summarised in Fig. 2.

### Primary outcomes

**Fig. 3:**
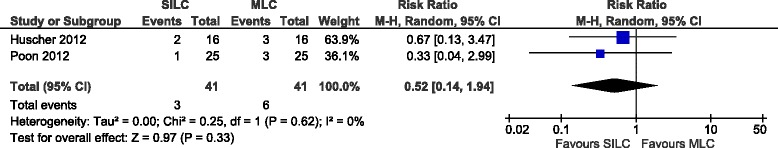
Forest plot, local complications (incl. intraoperative and postoperative)

There was no statistically significant difference observed in the pooled complication rates between the two treatment groups (RR = 0.52 (95 % CI 0.14 − 1.94), Fig. 3). In the study by Huscher et al. [23] one (6,3 %) major complication (anastomotic leakage, intraoperative bleeding) was reported for each intervention group and 1 minor complications (wound infection) in the SILC group and 2 minor complications (wound infections) in the MLC group. None of the reported complications by Poon et al. [19] were major complications (SILC group: one wound infection, MLC group: two wound infections and one ileus).

The mortality at longest available follow-up was not statistically significant between the two treatment groups in the study by Huscher et al. [23] (one (6,3 %) in each treatment group). The thirty-day mortality in this study was nil for both treatment groups, however, one patient in the SILC group died after 20 months of metastatic disease and one patient in the MLC group died after 12 months of a peritoneal carcinosis. There was no mortality in both treatment groups in the study by Poon et al. [19].

### Secondary outcomes

Due to the small number of included studies and events, we considered pooling data for only three (operative time, length of hospital stay and number of lymph nodes) of the 12 regarded secondary outcomes. For the other outcomes, the uncertainty about any estimated effect measure would be very high and its presentation might be misleading.

**Fig. 4:**
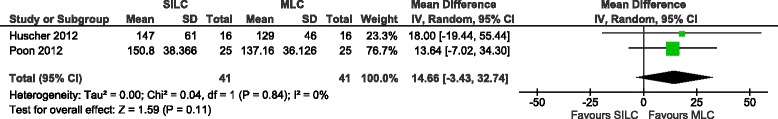
Forest plot, Operative time

**Fig. 5:**
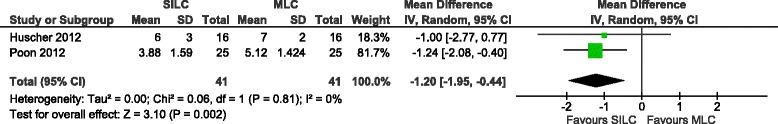
Forest plot, Length of hospital stay

**Fig. 6:**
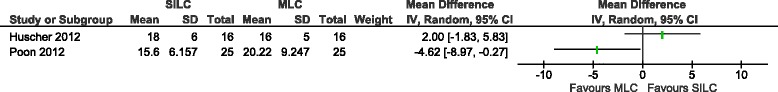
Forest plot, Number of lymph nodes harvested

While there was no statistically significant difference between SILC and MLC observed for the operative time (MD = +15 min (95 % CI -3 − 33), Fig. 4), the length of hospital stay was one day shorter in the SILC group (MD = -1 day (95 % CI -1.95 to -0.44), Fig. 5). There was substantial heterogeneity determined for the number of lymph nodes (*P* value = 0.03; I^2^ = 80 %, Fig. 6). Thus a pooled estimate for this outcome was not meaningful and no conclusions can be drawn from these data.

No significant differences were reported for the outcomes tumour-free margins [19, 23], conversion rate [19, 23], general complication [19, 23], estimated blood loss [19], bowel function or disease-free survival [23].

The reported postoperative pain intensity by Poon et al. [19] was presented without any adjustment for multiplicity. But even with a simple Bonferroni correction [66, 67] for all the compared pain scores, the day one result for the NRS pain score at rest remained statistically significantly different between the two treatment groups (SILC: nil (range 0−5), MLC: three (range 0−6), *P* value = 0.002).

None of the included studies examined cosmetic results, quality of life, or fatigue.

### Ongoing and unpublished trials

We identified 7 ongoing randomised trials comparing SILC and MLC through our additional search of two clinical trials registries (Table 4). We contacted the authors of trials with anticipated study completion date through 2017 to investigate the actual status of the trials and when we could expect their results to be available, but we either did not get any response or the author confirmed that they were still recruiting. Especially the last trial, due to its size of included participants, is very likely to have an important impact on our confidence in the estimate of effects and might even change the estimates.

**Table 4:** Ongoing randomised controlled trials

Registration number	Author	Country	Estimated completion date	Estimated enrollment *n*
NCT01626963	David W Borowski	UK	2016	50
NCT01203969	Suk Hwan Lee	Korea	2012	60
ISRCTN55622645	Weida Day	China	2012	100
NCT01959087	Yves Panis	France	2017	128
NCT02117557	Guoxin Li	China	2022	198
JPRN-UMIN000007220	Mitsuyoshi Ota	Japan	2020	200
NCT01480128	Hyung-Jin Kim	Korea	2017	388

## Discussion

We assessed 6 previous meta-analyses, which all tried to overcome the current sparseness of high-quality data by including observational studies such as case-matched studies. We assessed the methodological quality of these reviews and found it to be rather low. Therefore, the results and conclusions of these reviews may be affected by substantial methodological bias. Furthermore, we noticed that their reported results were inconsistent. While three reviews [10, 11, 15] reported that they could not find any difference concerning the conversion rate, one review [12] reported a higher conversion rate in the SILC group and another review reported a lower conversion rate in the SILC group [14]. We also noticed that several eligible studies were not included by several of these meta-analyses, although these primary studies were published at the time of literature search.

In the our systematic review and meta-analysis we identified two randomised controlled trials [19, 23], including 82 participants with malignant diseases, 41 in each of the two treatment. There were no patients with benign diseases included in this review. Based on these trials we found insufficient evidence to clarify whether single-incision laparoscopic colectomy (SILC) leads to less local complications (including both intraoperative and postoperative events) or lower mortality. Due to the small number of included studies, lack of event occurrence, as well as substantial heterogeneity in one outcome (number of lymph nodes harvested), meta-analyses were conducted only for two (operative time and length of hospital stay) of the 12 regarded secondary outcomes. Besides a significantly shorter hospital stay of one day, there was no statistically significant difference between SILC and MLC observed.

The total length of scar was not reported in either of the included studies. However, according to the description of the operation procedures, the reduction of total incision length in the SILC group is only a few centimetres. After adjustment for multiplicity, the reported postoperative pain intensity by Poon et al. [19] was statistically significantly reduced in the SILC group at the first day after the operation. The pain intensity of SILC was 1.64 points lower on average compared to the MLC with a 95 % confidence interval of 0.67 − 2.61. Since the confidence interval is relatively wide, it is not clear whether this difference is clinically important [68]. Since none of the studies reported cosmetic results, quality of life, or fatigue, and the reported reduction of pain intensity in the SILC group may be clinically unimportant, there is no evidence to investigate any potential dependency between post-operative comfort and total length of scars. Further randomised controlled trials are necessary to replicate reported results and to resolve inconsistencies between the studies.

The quality of the evidence was low, due to the sparse data and because the results from one of the two included studies were of a high risk of bias. Thus, the main limitation of this systematic review is the limited number of patients included in the meta-analysis for primary outcomes and very limited available results on secondary endpoints. However, this is not a limitation of our work but due to a lack of evidence and therefore not remediable.

The results of randomised and non-randomised studies sometimes differ [69] and non-randomised studies produce, on average, effect estimates that indicate more extreme benefits of the effects of health care than randomised trials [70], which is why it is not surprising that our review, including only RCTs, differs from these other reviews in some of the outcomes. Since these reviews predominantly present non-significant or heterogeneous results, our results are mostly in agreement with at least one of the reviews results. Thus, the inclusion of observational data does not lead to more reliable clinical recommendations, but instead leads to heterogeneity of results and increases risk of bias, due to the very low quality of the included studies. Clearly, more data are not necessarily better data. Therefore, our review presents the most reliable evidence currently available, by means of randomised controlled trials.

Although the available data are very sparse, so that there is a possibility that the lack of findings are due to a lack of evidence of effect and not due to a lack of effect itself, studies evaluating single incision laparoscopic surgery in different application areas showed similar results. A recent patient- and assessor-blinded randomised multi-centre trial [71], as well as a review including 659 patients from nine RCTs investigating single incision versus multi-incision laparoscopic cholecystectomy [72], could not show any benefit of single-incision laparoscopic cholecystectomy in postoperative pain, operating time, hospital stay and complication rate. The only significant benefit single-incision laparoscopic cholecystectomy showed in those studies was a better cosmetic result. Thus, one should consider the possibility that small advantages of SILC might be clinically irrelevant. Also, the available data enclosed in our review did not include a sufficient follow-up period to assess any long-term benefit or harm, so that a potentially negative effect of SILC cannot be excluded.

Although some surgeons will rate the present meta-analysis with only two included studies as not very valuable, it is important to describe how little data on SILC exist so far. Neither our review, including randomised controlled studies, nor the reviews, which also included observational studies, could confirm the safety. The lack of high-quality studies precludes a confirmation of safety, both in the short-term and in the oncological long-term. Nevertheless, surgeons increasingly practice SILC, which can be seen by the increasing number of published articles on this topic. The number of comparative studies published in the last four years has almost tripled [10, 11, 13–15], not counting the case reports and series published during this time. Hence, the main purpose of our systematic review is to remind the surgical community, currently deciding whether or not to use this new method that safety and effectiveness of SILC are yet to be confirmed. Also, this review acts as a warning sign that SILC should only be performed in a research setting. It is inevitable to wait for the results of further RCTs to be published.

## Conclusion

The currently available study results are too sparse to detect (or rule out) relevant differences between SILC and MLC. The quality of the current evidence is low, and the additional analysis of non-randomised data attempts, but does not solve this problem. For colorectal cancer patients, it is essential to assess oncologic outcomes (e.g. disease-free survival) in the long-term. For some complications (e.g. incisional hernia), a longer follow-up time is also necessary. SILC should still be considered as an experimental procedure, since the evidence of well-designed randomised controlled trials is too sparse to allow any recommendation.

## Abbreviations

AMSTAR: A Measurement Tool to Assess Systematic Reviews
ASA: American Society of Anesthesiologists
BMI: Body mass index
MD: Mean differences
MLC: Multi-port laparoscopic colectomy
RR: Risk ratio
SILC: Single-incision laparoscopic colectomy
TSC: Trial search coordinator
